# Treatment of pediatric patients with traumatic brain injury by Dutch Helicopter Emergency Medical Services (HEMS)

**DOI:** 10.1371/journal.pone.0277528

**Published:** 2022-12-30

**Authors:** Michelle Oude Alink, Xavier Moors, Pim de Bree, Robert Jan Houmes, Dennis den Hartog, Robert Jan Stolker

**Affiliations:** 1 Department of Anesthesiology, Erasmus University Medical Center-Sophia Children’s Hospital, Rotterdam, The Netherlands; 2 Helicopter Emergency Medical Services, Erasmus University Medical Center, Rotterdam, The Netherlands; 3 Intensive Care and Department of Pediatric Surgery, Erasmus University Medical Center-Sophia Children’s Hospital, Rotterdam, The Netherlands; 4 Department of Surgery-Traumatology, Erasmus University Medical Center, Rotterdam, The Netherlands; Monash University, AUSTRALIA

## Abstract

**Background:**

Sparse data are available on prehospital care by Helicopter Emergency Medical Service (HEMS) for pediatric patients with traumatic brain injury (TBI). This study focusses on prehospital interventions, neurosurgical interventions and mortality in this group.

**Methods:**

We performed a retrospective analysis of pediatric (0–18 years of age) patients with TBI treated by Rotterdam HEMS.

**Results:**

From January 2012 to December 2017 415 pediatric (<18 years of age) patients with TBI were included.

Intubation was required in in 92 of 111 patients with GCS ≤ 8, 92 (82.9%), compared to 12 of 77 (15.6%) with GCS 9–12, and 7 of 199 (3.5%) with GCS 13–15. Hyperosmolar therapy (HSS) was started in 73 patients, 10 with a GCS ≤8. Decompressive surgery was required in 16 (5.8%), nine patients (56.3%) of these received HSS from HEMS. Follow-up data was available in 277 patients. A total of 107 (38.6%) patients were admitted to a (P)ICU. Overall mortality rate was 6.3%(n = 25) all with GCS ≤8, 15 (60.0%) died within 24 hours and 24 (96.0%) within a week. Patients with neurosurgical interventions (N = 16) showed a higher mortality rate (18.0%).

**Conclusions:**

The Dutch HEMS provides essential emergency care for pediatric TBI patients, by performing medical procedures outside of regular EMS protocol. Mortality was highest in patients with severe TBI (n = 111) (GCS≤8) and in those who required neurosurgical interventions.

Despite a relatively good initial GCS (>8) score, there were patients who required prehospital intubation and HSS. This group will require further investigation to optimize care in the future.

## Background

Worldwide, traumatic brain injury (TBI) is a major cause of death and disability. In the Netherlands an incidence rate was found of 213.6 per 100.000 person years on average between 2010 and 2012. Young people (0–24 year olds) have an especially high incidence rate and disease burden, 268–270 per 100.000 person years [1]. A similar incidence rate and disease burden on young people was found in the United States and in Europe [2,3]. Traumatic brain injury (TBI) is defined as an alteration in brain function, or other evidence of brain pathology, caused by an external cause [4]. TBI is classified as severe, moderate or mild, based on the initial score on the Glasgow Coma Scale (GCS). A GCS of 3–8 is defined as severe TBI, 9–12 as moderate TBI and 13–15 as mild TBI [5]. Prehospital care for patients with TBI in the Netherlands is provided by the Emergency Medical Service (EMS), and if necessary assisted by the Helicopter Emergency Medical Service (HEMS) [6]. At this time data on prehospital interventions in pediatric patients with TBI by Dutch and international HEMS crews is scarce.

In 1995 the Dutch HEMS was initiated, which eventually led to a 24/7 nationwide HEMS availability in 2011 [7]. The main purpose of HEMS is to provide additional specialized emergency care on-scene and during transport to the hospital. Patient transportation by helicopter is less common, mainly due to short distances to a suitable (level 1 or level 2 trauma center) hospital in the Netherlands [8].

An important part of the care for (pediatric) patients with TBI is prehospital assessment and, when needed, interventions by EMS and HEMS crews. Prehospital interventions are aimed at prevention of secondary brain injury by airway management, adequate ventilation, fluid resuscitation and the prevention of cerebral herniation and edema [9,10]. In patients with TBI the Dutch EMS protocol is limited to fluid resuscitation, oxygen administration and they can elevate the head 30 degrees [11]. Pain medication is only an option with a GCS ≥ 10, due to risk of hypoventilation.

The HEMS crew can also provide anesthesia, monitor and control EtCO2 by ventilation, administer hyperosmolar therapy and administer medication to regulate blood pressure.

Previous studies have shown that prehospital interventions by a physician-staffed HEMS decreases prehospital hypoxia and increases the number of secured airways in patients with severe TBI, which may contribute to an improved neurological outcome [12].

Treatment of TBI continues in-hospital, where in addition to sedation and hyperosmolar therapy cerebrospinal fluid (CSF) drainage or decompressive surgery are possible therapeutic options [13].

The purpose of this study is to get a better understanding of the prehospital HEMS interventions, in-hospital neurosurgical interventions and mortality in pediatric patients with traumatic brain injury in the Netherlands. Questions that need to be adressed are: What pediatric patients with TBI have a higher risk of mortality? What HEMS / EMS and in-hospital interventions can be applied to reduce this mortality risk? This could identify patients at high risk for interventions and mortality and possibly adjust HEMS dispatch criteria and/or training to cater to high risk patients in the future.

## Methods

### Setting

EMS had over 1.3 million dispatches in 2016, on an estimate Dutch population of 16.98 million [14].

All Dutch Emergency Medicine Service (EMS) crews consist of a specialized nurse with extensive training in pre-hospital emergency care of nine months. Most have started as an emergency department nurse, ICU or anesthesia nurse. They are assisted by an ambulance driver whom is trained to assist the nurse during medical procedures such as advanced life support.

Dutch EMS provides basic or advanced life support when dealing with injured or ill patients from all ages. The EMS crew works according to standard national protocols, describing when and in what cases procedures can be performed and/or medication can be administered [11].

EMS dispatchers use strict dispatch protocols to determine when to dispatch one of the HEMS crews. The HEMS crew can be dispatched primarily, based on information given in the initial distress call. This is based on the mechanism of injury or patient’s vital signs as provided by the distress caller. Another option is secondary dispatch, when the HEMS crew is requested by EMS during patient assessment. EMS crews can also decide to cancel the HEMS dispatch according to the predetermined cancel criteria [15].

There are four HEMS regions, divided over the Netherlands to provide coverage of the entire country. The HEMS crew in the Netherlands consists of either an anesthesiologist or specially trained trauma surgeon, assisted by a specialized nurse and a pilot. Both a helicopter and a rapid response vehicle are available for transport. The ground vehicle may be opted when weather conditions restrict helicopter flights, when ground transport is the fastest way to reach the patient, when the incident is nearby or when the helicopter requires maintenance.

### Data collection

This study comprises a retrospective chart review. During a dispatch all patient characteristics, administered medication and applied interventions were registered manually by the attending HEMS physician, either on paper or in a newly setup database, accessible by tablet. Data was collected of all pediatric patients, <18 years of age, treated by the Rotterdam HEMS between 1st of January 2012 and 31 December 2017. HEMS dispatches cancelled before patient contact are excluded. Specific content was not obtained from parents or guardians, as data was sufficiently anonymised. The study’s setup for data collection and processing was approved by the Medical Ethical Committee of the Erasmus University Medical Center Rotterdam.

### Ethics approval

The Medical Ethical Committee of the Erasmus University Medical Center Rotterdam (MEC-2018-1021) approved this study.

### Inclusion/Exclusion criteria

We selected all trauma patients that were classified by the HEMS physician or Emergency Department as traumatic brain injury of all severities.

Included diagnoses were concussion, brain contusion, intracranial hematomas, skull fractures and diffuse axonal injuries.

Patients with GCS <15 without traumatic brain injury were excluded, for example; seizures, increased intracranial pressure due to tumors, spontaneous intracranial hemorrhage and intoxications.

### Variables

All included dispatches were reviewed on the patient demographics, the mechanism of injury, prehospital interventions, administered medication, transport to the hospital, in-hospital treatment and mortality.

Hospital records were obtained for patients admitted to the Erasmus University Medical Center Rotterdam–Sophia Children’s hospital concerning neurosurgical interventions during their hospital stay and mortality. This hospital is the designated neuro trauma center for pediatric patients in the region, therefore the majority of patients were treated here. A small number of patients were transported to other (neuro) trauma centers. Follow up data on in-hospital interventions is missing in those cases, so our analysis of these interventions is limited to data of patients transported to the Erasmus University Medical Center Rotterdam–Sophia Children’s hospital.

From the Dutch Population Register (Basisregistratie Personen), we requested additional data on the mortality of patients whose death could not be confirmed from initial assessment or hospital records. Follow up of the mortality varied from three months to six years and three months. Primary outcome parameters of our study were mortality, prehospital interventions and in-hospital neurosurgical interventions.

All data was analyzed using descriptive statistics in IBM SPSS Statistics 24.0 (IBM, New York, USA).

## Results

### Inclusion and exclusion

**Fig 1:**
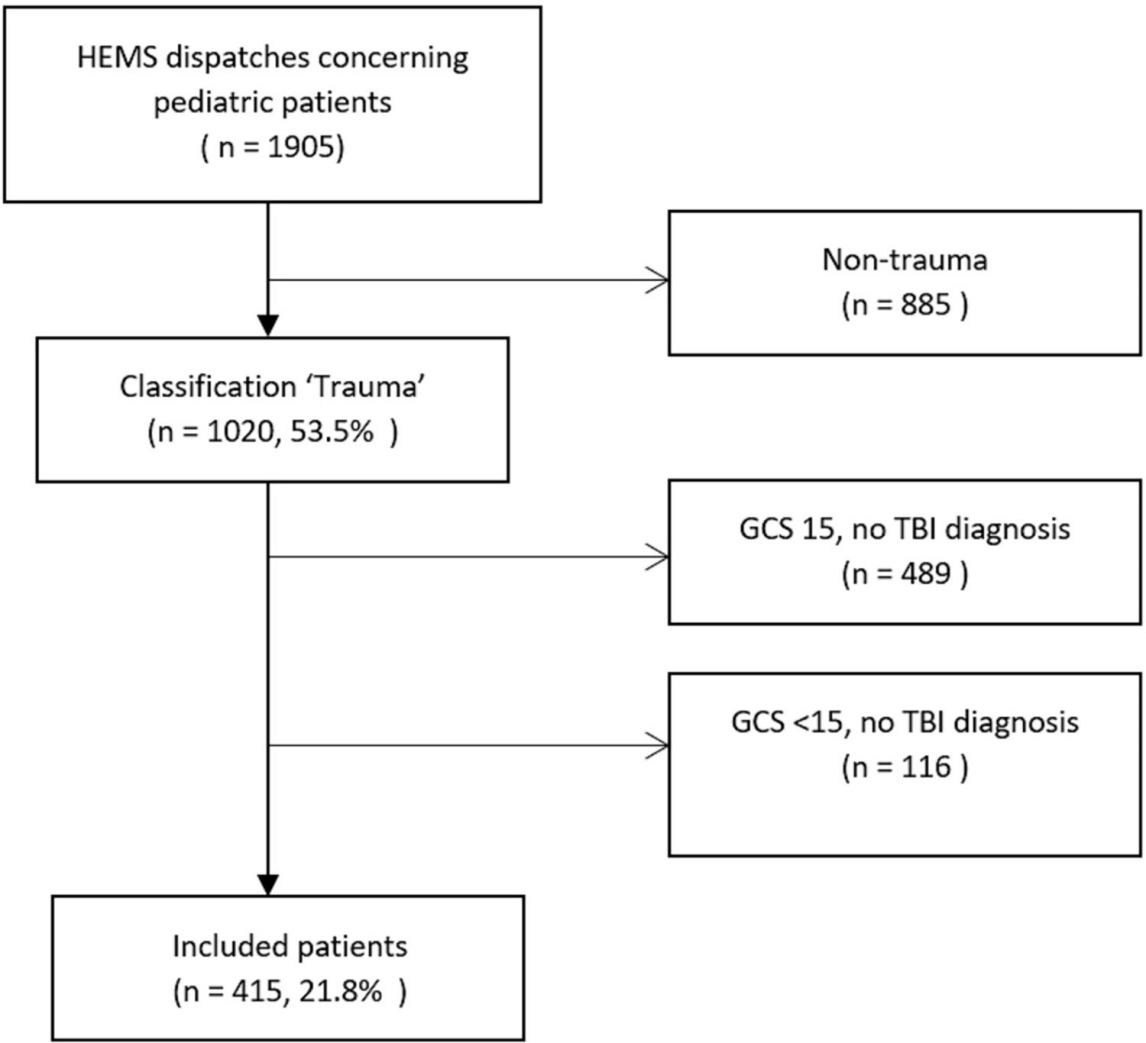
Inclusion/Exclusion chart.

From the 1^st^ of January 2012 until 31 December 2017 the Rotterdam HEMS was dispatched 1905 times concerning pediatric patients. After exclusion 415 (21.8%) patients with TBI were included (Fig 1).

### Demographics

Out of all TBI patients, 111 (26.7%) were classified as severe TBI, 77 (18.6%) had a moderate TBI and 199 (47.8%) had mild TBI. In 28 (6.7%) patients the initial GCS score was not documented.

Average age was 7.9 years; the range 0–18 years. In 49 patients the date of birth was unknown, in this case the HEMS physician filled in an ‘age estimate’, which was used in the analysis. Estimated age in years was used to calculate the mean age and to divide all patients into age groups.

Of all pediatric patients with traumatic brain injury 258 (62.0%) were male. In one patient no gender was documented.

In the severe TBI group, 69 patients (62.2%) were male and 42 female (37.8%). Of all patients with moderate TBI 56 (72.7%) were male and 20 (26.0%) female. 116 male patients (58.3%) and 83 female patients (41.7%) were classified as mild TBI. In 17 males and 11 females no GCS was registered (Table 1).

**Table 1:** Demographics, mechanism of injury, EMS/HEMS interventions, transport, follow up in hospital and mortality of pediatric patients with TBI.

	n			
Variable	Overall	GCS ≤ 8	GCS 9–12	GCS 13–15
Total Gender (n = %) • Male • Female • Unknown	415 • 258 (62.2%) • 156 (37.6%) • 1 (0.2%)	111 • 69 (62.2%) • 42 (37.8%) • 0	77 • 56 (72.7%) • 20 (26.0%) • 1 (1.3%)	199 • 116 (58.3%) • 83 (41.7%) • 0
Age mean	• 7.9	• 9.7	• 8.9	• 7.1
Mechanism of injury • Traffic accident • Fall from height • Violence • Self-inflicted • Other	431 • 173 (40.1%) • 217 (50.3%) • 8 (1.9%) • 5 (1.2%) • 29 (6.7%)	118 • 68 (57.6%) • 33 (30.0%) • 5 (4.2%) • 3 (2.5%) • 9 (7.6%)	79 • 33 (41.8%) • 43 (54.4%) • 0 • 0 • 3 (3.8%)	205 • 63 (30.7%) • 123 (60.0%) • 2 (0.98%) • 2 (0.98%( • 15 (7.3%)
EMS^1^ procedures • Intubation • Medication • Paracetamol • Benzodiazepines • Fentanyl • Ketamine • Adrenaline • Other HEMS^2^ procedures • Intubation • Medication ○ Analgesia ▪ Paracetamol ▪ Fentanyl ▪ Esketamine ○ Benzodiazepines ○ Propofol / Etomidate ○ Rocuronium / Succinylcholine ○ Adrenaline ○ Atropine ○ Tranexamic Acid ○ Hyperosmolar therapy • Blood transfusion	• 10 (2.4%) • 9 (2.2%) • 15 (1.2%) • 19 (4.6%) • 3 (0.7%) • 7 (1.7%) • 20 (4.8%) 111 (26.7%) • 29 (7.0%) • 150 (36.1%) • 21 (5.1%) • 95 (22.9%) • 89 (21.4%) • 91 (21.9%) • 13 (3.1%) • 7 (1.7%) • 12 (2.9%) • 73 (17.6%) • 8 (1.9%)	• 10 (9.0%) • 0 • 5 (4.5%) • 2 (1.8%) • 0 • 7 (6.3%) • 6 (5.4%) • 92 (82.9%) • 2 (1.8%) • 63 (56.8%) • 13 (11.7%) • 56 (50.5%) • 64 (57.7%) • 74 (66.7%) • 12 (10.9%) • 7 (6.3%) • 10 (9.0%) • 62 (55.9%) • 8 (7.2%)	• 0 • 0 • 5 (6.5%) • 4 (5.2%) • 1 (1.3%) • 0 • 2 (2.6%) • 12 (15.6%) • 5 (6.5%) • 34 (44.2%) • 0 • 23 (29.9%) • 15 (19.5%) • 10 (13.0%) • 0 • 0 • 1 (1.3%) • 8 (10.4%) • 0	• 0 • 8 (4.0%) • 5 (2.5%) • 11 (5.5%) • 1 (0.5%) • 0 • 10 (5.0%) • 7 (3.5%) • 16 (8.0%) • 49 (24.6%) • 7 (3.5%) • 15 (7.5%) • 10 (5.0%) 6 (3.0%) • 0 • 0 • 1 (0.5%) • 2 (1.0%) • 0
Transport • Deceased on-scene • Hospital with PICU^3^ and neurosurgical care • Hospital without PICU, with neurosurgical care • Hospital without PICU and no neurosurgical care • Unknown / transport unknown Transport by • EMS transport • HEMS transport ○ HEMS ground transport ○ HEMS air transport • Transport unknown	• 8 (1.9%) • 308 (74.2%) • 32 (7.7%) • 50 (12.0%) • 17 (4.1%) • 157 (37.8%) • 248 (59.8%) • 230 (55.4%) • 18 (4.3%) • 2 (0.5%)	• 8 (7.2%) • 91 (82.0%) • 7 (6.3%) • 2 (1.8%) • 3 (2.7%) • 3 (2.7%) • 100 (90.1%) • 89 (80.2%) • 11 (9.9%) • 0	• 0 • 64 (82.1%) • 8 (10.4%) • 3 (3.9%) • 2 (2.6%) • 15 (19.5%) • 62 (80.5%) • 60 (77.9%) • 2 (2.6%) • 0	• 0 • 128 (64.3%) • 15 (7.5%) • 44 (22.1%) • 12 (6.0%) • 124 (62.3%) • 73 (36.7%) • 68 (34.2%) • 5 (2.5%) • 2 (1.0%)
• Follow-up ER ○ Deceased in ER^4^ ○ Direct OR^5^ ○ PICU ○ High Care ○ Regular ward ○ Discharge from ER ○ Transfer ward other hospital ○ Transfer (P)ICU other hospital • Neurosurgical interventions ○ ICP^6^ pressure monitor ○ ELD^7^ ○ EVD^8^ ○ Decompressive craniectomy	277 • 1 (0.4%) • 27 (9.7%) • 107 (38.6%) • 2 (0.7%) • 86 (31.0%) • 22 (7.9%) • 30 (10.8%) • 2 (0.7%) • 50 (18.1%) • 43 (15.5%) • 2 (0.7%) • 7 (2.5%) • 16 (5.8%)	84 • 1 (1.2%) • 17 (20.2%) • 57 (67.9%) • 0 • 1 (1.2%) • 3 (3.6%) • 5 (6.0%) • 0 • 40 (47.6%) • 37 (44.0%) • 1 (1.2%) • 6 (7.1%) • 12 (14.3%)	56 • 0 • 3 (5.4%) • 27 (48.2%) • 0 • 17 (30.4%) • 3 (5.4%) • 6 (10.7%) • 0 • 5 (8.9%) • 4 (7.1%) • 0 • 1 (1.8%) • 2 (3.6%)	116 • 0 • 6 (5.2%) • 16 (13.8%) • 1 (0.8%) • 57 (49.1%) • 16 (13.8%) • 18 (15.5%) • 2 (1.7%) • 4 (3.4%) • 1 (0.9%) • 1 (0.9%) • 0 • 2 (1.7%)
• Mortality ○ On-scene ○ Day of dispatch ○ Mean in days, since day of dispatch ○ Range in days	25 (6.3%) • 8 (32.0%) • 15 (60.0%) • 1.16 • 0–8	25 (22.5%) • 8 (32.0%) • 15 (60.0%) • 1.16 • 0–8	0	0

^1^ EMS: Emergency Medical Services.

^2^ HEMS: Helicopter Emergency Medical Services.

^3^ Pediatric Intensive Care Unit.

^4^ Emergency Room.

^5^ Operating Room.

^6^ Intracranial pressure.

^7^ External lumbar drain.

^8^ External ventricular drain.

### Mechanism of injury

Traffic accidents (40.0%) and falls from height (50.2%) are the main injury mechanism.

In 16 patients there is an overlap between injury mechanisms, for example a fall after a traffic accident or a self-inflicted stab- or gunshot wound.

When looking at patients with severe TBI, traffic accidents comprise 57.6%. Patients with moderate or mild TBI more often suffered from falls, 54.4% and 60.0%, respectively.

**Fig 2:**
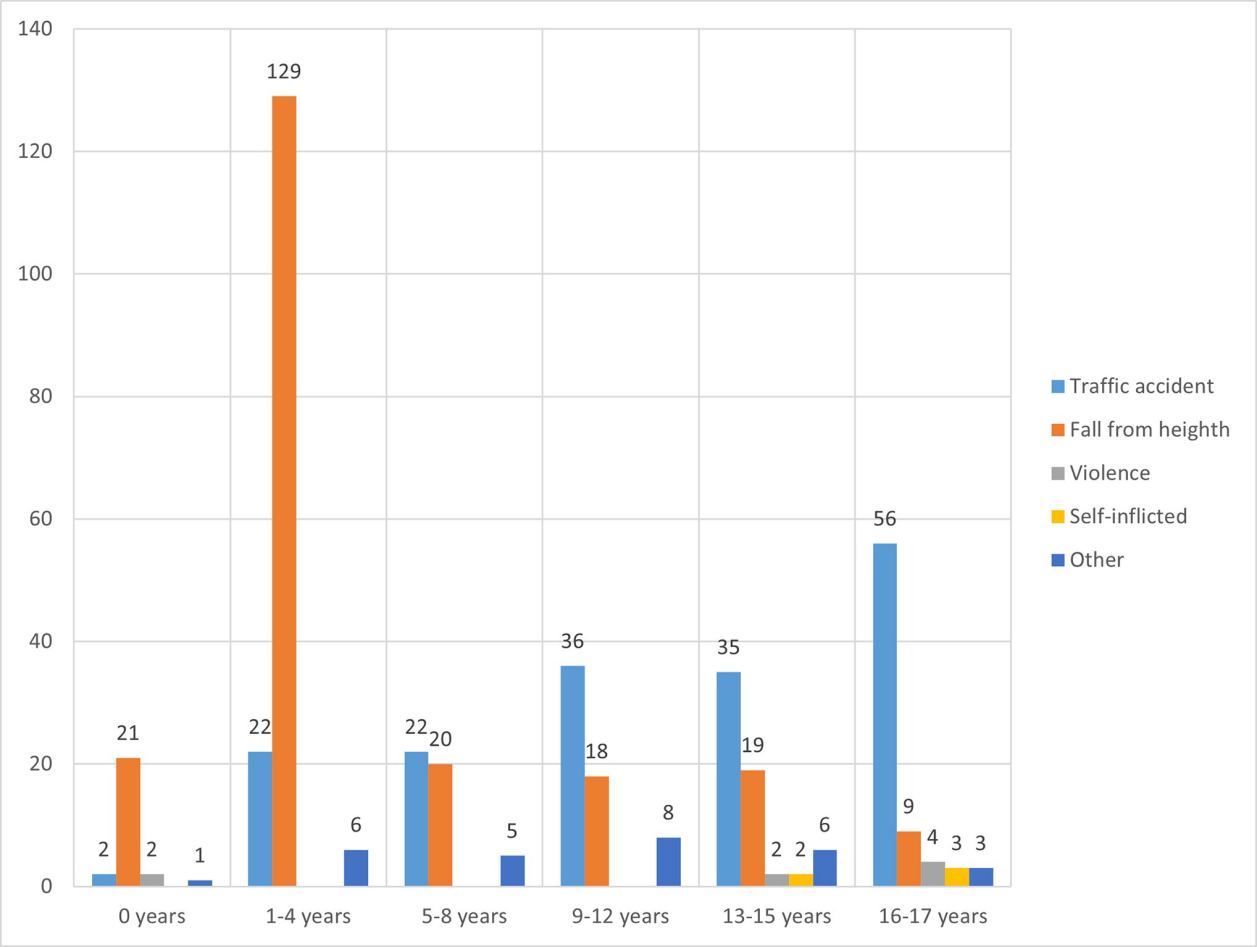
The mechanism of injury in comparison with age group of pediatric patients with TBI.

In Fig 2 the mechanism of injury is contrasted with the patients age. Of all 154 patients between 1–4 years old, 129 TBI cases (83.8%) occurred after a fall from height. Of these patients, 45 (34.9%) fell from a flight of stairs. In patients between 13–15 and 16–17 years old, the majority of injuries was the result of a traffic accident, 59.3% and 78.9%. Of the 16–17 years old patients involved in a traffic accident 21 (37.5%) were on a motor scooter, 12 (21.4%) were riding a bicycle, it was not registered whether they were wearing a helmet. In the Netherlands it is uncommon to wear a helmet on a bicycle and it is also not obligatory on a scooter with a maximum of 25km/hour. It is legal to ride a 25km/hour from 16 years of age.

### Intubation

A total of 111 (26.7%) patients required intubation by HEMS. Another ten (2.4%) patients were intubated by EMS before the arrival of the HEMS crew, all had a GCS of 3, and 8 of these patients required CPR at the moment of intubation. Of 111 patients with an initial GCS score ≤8, 92 (82.9%) required intubation. Another 12 (15.4%) patients with an initial GCS score of 9–12 and seven (3.5%) patients with an initial GCS score of 13–15 were intubated. Of these seven patients with a GCS score of 13–15, two patients were intubated because of vomiting, two patients after a sudden GCS decrease, two patients because of agitation and one because of multiple injuries. In 12 cases intubation was performed during cardiopulmonary resuscitation (CPR). Of these 12 patients 11 (91.6%) died, the last patient was lost to follow up.

### Medication

EMS administered paracetamol, fentanyl, benzodiazepines (off-protocol) and ketamine, but in much fewer patients than HEMS, as seen in Table 1. In 20 (4.8%) patients another type of medication was given, mostly antiemetics. Adrenaline was administered in seven (1.7%) patients, all during CPR. No medication was administered by EMS in 317 (76.8%) patients, while HEMS administered no medication in 172 (41.6%) patients.

HEMS administered adrenaline in 13 (3.1%) patients, in 11 patients during CPR and in two patients as part of treatment for a possible hypovolemic shock. Another seven (1.7%) patients received atropine for bradycardia. Intravenous hypnotics, propofol or etomidate, were administered in 89 (21.4%) patients. During an intubation procedure, apart from sedative medication, in 91 (21.9%) patients a muscle relaxant, rocuronium or succinylcholine, was administered.

HEMS physicians also administered medication in order to manage suspected increased intracranial pressure (ICP). A total of 73 (17.6%) patients received mannitol, HSS (hypertonic saline solution) or both. Prehospital blood transfusion was given to eight (1.9%) patients, all after traffic accidents, which led to severe blood loss and/or suspected shock.

### Transport

Out of 415 patients, 157 (37.8%) were transported by EMS alone and 248 (59.8%) were accompanied by HEMS, of which 230 (55.4%) by ground transport and 18 (4.3%) by helicopter. Air transport was chosen mainly for logistic reasons (time to hospital was shorter). Eight (1.9%) patients died on-scene. Of two (0.5%) patients the means of transportation are unknown. Patients with severe TBI were transported by helicopter more often compared to patients with moderate or mild TBI, as seen in Table 1.

A total of 309 (74.3%) patients were transported to a hospital with a Pediatric Intensive Care Unit (PICU), of which 273 (65.4%) to the Erasmus University Medical Center Rotterdam–Sophia Children’s Hospital. Another 32 (7.7%) patients were transported to a hospital with 24/7 available neurosurgery (NCH) but no PICU and 50 (12.0%) to a hospital with neither PICU nor neurosurgeon on call.

Both in the severe and moderate TBI group the majority of patients were transported to a hospital with PICU and NCH, 91 (82.0%) and 65 (83.3%) patients respectively. In the mild TBI group 128 (64.3%) patients were transported to a PICU and/or NCH hospital.

Of the 308 patients transferred to a PICU and/or NCH hospital, 206 (66.9%) were transported by HEMS ground vehicle and 18 (5.8%) by helicopter. Out of the 50 patients transferred to a regular hospital, only 4 were transported by HEMS ground transport. The remaining 46 were transported by EMS.

### Neurosurgical interventions/follow-up

In hospital follow-up data was available in 277 patients, of which 272 were transported to the Erasmus University Medical Center Rotterdam–Sophia Children’s hospital. After initial assessment in the ER 27 (9.7%) patients went for immediate surgery. During their initial hospital stay 50 (18.1%) out of 277 patients underwent neurosurgical interventions. In total 43 (15.5%) patients received an ICP pressure monitor, two (0.7%) received a lumbar drain and eight (2.9%) an external ventricular drain. Decompressive uni- or bilateral surgery was required for 16 (5.8%) patients. A total of 107 (38.6%) patients were admitted to the (P)ICU, 2 (0.7%) patients to the High Care unit and 86 (31.0%) patients went to a regular ward. Another 21 (7.7%) patients were discharged home from the ER and 31 (11.3%) were transferred to a different hospital, of which 1 to another (P)ICU.

The majority of neurosurgical interventions were performed on patients with severe TBI.

Of the 84 patients with severe TBI whose follow up data was registered, 40 (47.6%) required neurosurgical interventions. Of all patients with severe TBI, 57 (67.9%) were admitted directly to the PICU and another 17 (20.2%) underwent surgery before being admitted to the PICU. Only 3 (3.7%) patients with severe TBI based on the initial GCS were discharged from the ER directly after rapid improvement, and 6 (7.3%) were admitted to a regular ward. One (1.2%) patient died whilst being treated in the ER.

### Mortality

Overall mortality was 25 (6.3%) from the 277 patients with available follow up data. Eight patients were declared dead on scene by HEMS. No mortality occurred in patients with a GCS >8. Of all 25 mortalities 15 (60%) occurred within 24 hours, and all but one, died within a week (Table 1). The average time until mortality was 1.16 days after dispatch. Afterwards the Dutch population register (GBA) provided information on 273 out of 415 patients, wherein no further deaths were found.

Of 277 patients with available in-hospital follow up, 50 required neurosurgical interventions (Table 1).

All mortalities were found in the severe TBI group of 40 patients, were 9 patients (22.5%) died.

In this high mortality group, patients that required an insertion of an ICP pressure monitor, extraventricular drain (EVD) or decompressive surgery had an even higher mortality rate, respectably 22.9 and 25.0%. Table 2 shows patients with the highest mortality rate required neurosurgical interventions.

**Table 2:** Mortality associated with neurosurgical interventions in-hospital.

All neurosurgical interventions	9/40 (22.5%)
ICP monitor insertion	8/35 (22.9%)
ELD insertion	0
EVD insertion	2/6 (33.3%)
Decompressive surgery	3/12 (25.0%)

## Discussion

The GCS is used to classify the severity of TBI in pediatric patients. It is difficult to provide an accurate GCS during initial assessment of (young) children, especially in a stressful pre-hospital setting.

Furthermore, GCS is a dynamic variable in trauma patients which means the initial classification of TBI can worsen or improve. Nevertheless GCS is still the best scoring system and has an adequate predictive value [16–18].

The majority of intubations was necessary in the group with a low GCS, as supported by literature [19]. Although not expected, we found seven intubations in the mild TBI (GCS 13–15, n = 199) group, due to vomiting, deterioration in GCS, agitation and multiple injuries. This means interventions are rarely performed when a patient has a GCS 13–15, but HEMS is still needed in a minority of the cases for airway management, hemodynamic assistance or additional pain relief. Only relying on a high initial GCS is therefore no correct cancel criterion for HEMS dispatch. Patients must be monitored and a thorough continuous assessment must be performed to identify patients in need for HEMS support.

Limited exposure to severe pediatric trauma by EMS crews may still lead to suboptimal prehospital care for severely injured pediatric patients [20]. In a large cohort study from Germany, pediatric emergencies accounted for only 6.3% of all EMS and 8.5% of all HEMS cases. In all pediatric patients with TBI, German HEMS was present in 73.9% of the time. HEMS-attended cases received more advanced interventions [21]. This higher exposure to pediatric TBI lead HEMS crews with more experience than regular EMS crews. In the Netherlands, a study showed HEMS is dispatched in over half of the patients with TBI, and patients treated by HEMS were younger and had a higher injury severity [22]. This is possibly due to the dispatch criteria for the injured child.

A large cohort study from the USA found 6.8% of all EMS dispatches involved pediatric patients, and in just 1% of those dispatches critical procedures were performed. Intubation, defibrillation, needle decompression and intraosseous catheter insertion are examples of critical procedures. An overall success rate of 76.2% was found for pediatric intubations [23]. A study from Belgium shows a success rate of 92.4% for pediatric intubations in severely ill patients when performed by physician-staffed EMS teams [24].

In our study only ten (2.4%) patients are intubated by EMS and 111 (26.7%) by HEMS.

A Dutch study shows that experience in endotracheal intubation significantly improves survival rate in patients with severe TBI [25]. For a long time there has been an ongoing discussion on whether pediatric intubation should be completely removed from the national EMS protocol, due to the high rate of complications: esophageal intubations, inappropriately sized uncuffed tubes and even lethal ventilator settings (administered settings >300% of recommended settings) [26]. The latest nationwide EMS protocol (2016) prohibits intubation in patients with TBI [11].

EMS administered S-ketamine in only three (0.7%) patients and HEMS in another 21 (5.1%) patients.

For a long time, there have been concerns on the use of ketamine in patients with traumatic brain injury. Intravenous ketamine would increase cerebral blood flow and therefore intracranial pressure [27,28]. More recent studies show ketamine actually has neuroprotective abilities, as it could decrease ICP (in children) with intracranial hypertension [29–31]. Therefore we may consider the use of S-ketamine a safe option for patients with TBI.

A total of 73 (17.6%) patients received hyperosmolar therapy to manage an increase in intracranial pressure. In the latest guidelines, a therapy which has been proven useful in recent literature, and without any serious side effects. Data on usage of hyperosmolar therapy in pediatric patients is scarce. A favourable effect on intracranial pressure was however found in some studies with small patient groups [32–34]. However, in our study only nine of 16 patients who received decompressive surgery were treated with hyperosmolar therapy. Estimating if patients require hyperosmolar therapy out-of-hospital appears to be difficult, and sometimes other procedures are more necessary to stabilize the patient before arriving to the hospital. HEMS physicians could consider to administer hyperosmolar therapy in a broader range of patients, since no side effects are known in literature and almost half of decompressive surgery patients didn’t receive the treatment".

A systematic review on pediatric TBI epidemiology found a mortality rate between 1–7% [35].

A study from England, also in an urban environment with an HEMS service, found an 8% mortality in patients with TBI of all severities, as confirmed by a CT scan [36]. In our study we found an overall mortality of 6.3%. All deceased patients in our study suffered from severe TBI. In that particular group the mortality rate was 22.5%. Of all 25 deceased patients, 24 died within 7 days after the incident. This high proportion of early mortality is in accordance with the literature [37,38].

In the literature we found a need for neurosurgical interventions in 12.9% of the pediatric patients with severe TBI, 1.8% of patients underwent decompressive surgery [39]. Both severe TBI and the requirement for neurosurgical interventions are associated with a high(er) mortality risk. Patients with severe TBI requiring decompressive surgery or insertion of an EVD have the worst prognosis.

In the nationwide HEMS dispatch and cancel criteria overtriage is deemed acceptable up to 50%, in order to lower the undertriage rate below 10% [7]. In our study HEMS complies with these criteria, as they assisted during transport in almost 60% of the cases (Table 1), and even in the best performing 50% of the patients (GCS 13–15) interventions were performed by HEMS physicians. Of all a priori cancelled dispatches the patients age and mechanism of injury/disease is unknown, so determining over- and undertriage ratio’s in our patient cohort is unreliable.

Obviously, attention should be focused on pediatric patients with severe TBI, due to the high mortality rate. But our study shows that even patients with an initial good GCS may require HEMS interventions, due to a sudden deterioration, agitation or multiple injuries.

Further research is needed on patients with moderate or mild TBI. It is possible EMS would be able to manage mildly injured pediatric TBI patients without HEMS dispatch, and to recognize patients that require HEMS and possibly neurosurgical in-hospital interventions.

Previous studies found a majority of both overall trauma and TBI patients to be male, similar to our cohort [2,20]. Our findings that different age groups suffer from different injuries (Fig 2) are also supported by literature [40]. Patients between one and four years old tend to get injured mainly in falls from height, older children mainly in traffic accidents.

### Strengths and limitations

Patients were included when the HEMS physician established a prehospital diagnosis of brain injury.

In severely injured multi-trauma patients, a brain injury diagnosis may be overlooked.

When a patient suffers from traumatic amputations or massive external or internal hemorrhages and dies on-scene it is possible the HEMS physician did not record a neurological diagnosis, due to more severe injuries that required immediate treatment.

Another limitation could be that patients who were given no brain injury diagnosis were transported to a regular hospital, and after a clinical assessment by a physician were given a TBI diagnosis.

The follow-up data of all patients who travelled to these smaller regular hospitals is often unavailable. Therefore, it is possible some patients with a later established TBI diagnosis may have been excluded wrongfully.

Another major limitation of our study is the retrospective character. All data was entered manually by the HEMS physician during or shortly after the initial dispatch. Any unknown or not entered data was therefore lost immediately afterwards. This adds the need for a more robust electronic data manager, where the HEMS physician can enter the patients parameters immediately and electronically on-site.

The above limitation applies to the absence of data on time and distance covered during the initial dispatch, as well as time to the nearest (neurosurgical) centre. New(er) studies should look at including time to reach definitive care as a parameter, as it could be beneficial to see the effect this has on final outcome(s).

Because of differences in the organization of prehospital emergency care, comparison between studies from different countries remains difficult.

To our knowledge not such a large cohort of pediatric TBI patients has been reviewed up to now.

Where most TBI studies focus on patients with severe TBI, our study has included patients with all GCS scores. Even in patients with mild and moderate TBI prehospital interventions were required. These results show patients with an initial GCS score >8 should still require close monitoring in case of sudden deterioration.

## Conclusions

The Dutch HEMS provides essential emergency care for pediatric TBI patients, by performing medical procedures outside of regular EMS protocol. Mortality was highest in patients with severe TBI (GCS≤8) and in those who required neurosurgical interventions.

Despite a relatively good initial GCS (>8) score, there were patients who required prehospital intubation and HSS. This group will require further investigation to optimize care in the future.
